# Dyadic influences on awareness of condition in people with dementia: findings from the IDEAL cohort

**DOI:** 10.3389/fnagi.2023.1277336

**Published:** 2023-12-11

**Authors:** Catherine M. Alexander, Anthony Martyr, Laura D. Gamble, Catherine Quinn, Claire Pentecost, Robin G. Morris, Linda Clare

**Affiliations:** ^1^The Centre for Research in Ageing and Cognitive Health, University of Exeter Medical School, Exeter, United Kingdom; ^2^NIHR Applied Research Collaboration South West Peninsula, Exeter, United Kingdom; ^3^Population Health Sciences Institute, Newcastle upon Tyne University, Newcastle, United Kingdom; ^4^Centre for Applied Dementia Studies, University of Bradford, Bradford, United Kingdom; ^5^Wolfson Centre for Applied Health Research, Bradford, United Kingdom; ^6^Department of Psychology, Institute of Psychiatry, Psychology and Neuroscience, King's College London, London, United Kingdom

**Keywords:** discrepancy, informant ratings, everyday difficulties, dyadic, caregiver stress, carer stress

## Abstract

**Introduction:**

The discrepancy between caregiver-ratings and self-ratings of abilities is commonly used to assess awareness in people with dementia. We investigated the contribution of caregiver and dyadic characteristics to the difference in perspective between caregiver-informants and people with dementia about difficulties experienced, when considering awareness of condition.

**Methods:**

We conducted exploratory cross-sectional analyses using data from the IDEAL cohort. Participants were 1,038 community-dwelling people with mild-to-moderate dementia, and coresident spouse/partner caregivers. The Representations and Adjustment to Dementia Index (RADIX) checklist reporting difficulties commonly experienced in dementia was completed by 960 caregiver-informants and 989 people with dementia. Difference in scores was calculated for 916 dyads. Demographic information, cognition, informant-rated functional ability and neuropsychiatric symptoms were recorded for the person with dementia. Self-reported data were collected on mood, comorbidity, religion, importance of religion, relationship quality, and caregiver stress.

**Results:**

For most dyads, caregivers reported more RADIX difficulties than people with dementia. Caregiver RADIX ratings were more closely associated with informant-rated functional ability and neuropsychiatric symptoms than with cognition. More RADIX difficulties and higher stress were reported by female caregivers. Greater RADIX difference was associated with more caregiver stress, and older age but less depression in people with dementia.

**Conclusion:**

Few dyadic characteristics were important, but caregiver stress was higher where caregivers reported more RADIX difficulties and/or the difference in perspective was greater, whereas partners with dementia reported better mood. In addition to offering information about awareness of condition, the caregiver rating and difference in perspectives could indicate where more support is needed.

## Introduction

Dementia is an increasingly prevalent condition, with global numbers predicted to rise to 139 million by 2050 (Alzheimer’s Disease International, 2023). People with dementia vary in their awareness of the condition, in particular understanding their own symptoms and diagnosis, and the implications of these for themselves and others (Clare, 2004; Mograbi et al., 2012; Clare et al., 2012a). The degree of awareness can influence the experience of living with dementia, including the well-being of the person with dementia (Aalten et al., 2005; Azocar et al., 2021; Alexander et al., 2021a), and the well-being of the caregiver (Dourado et al., 2016; Perales et al., 2016). It can also affect safety, e.g., in driving (Cotrell and Wild, 1999) and other everyday activities (Parrao et al., 2017). For clinicians and organizations providing supportive care, awareness is additionally relevant as lack of awareness can affect clinical communication (Dooley et al., 2015), influence decisions about health care treatments (Karlawish, 2008), and increases the cost of care (Turró-Garriga et al., 2016).

To assess awareness in dementia, measures commonly use the discrepancy between ratings of ability made by the person with dementia and the caregiver as informant (Alexander et al., 2021b). This approach assumes that the caregiver rating is more accurate, and any divergence indicates differing degrees of awareness in the person with dementia. This can vary depending on the domain being evaluated, e.g., memory, functional ability, socioemotional ability (Clare et al., 2012a; Alexander et al., 2021b). Accordingly, it is important to understand the basis for the caregiver rating in different domains, to clarify which factors contribute to the caregiver rating and to differences in perspective. Possible bias in caregiver ratings should be acknowledged and may be influenced by other issues affecting the caregiver or other features relating to the person with dementia (Clare, 2004). Caregiver ratings are sometimes less accurate and indicate more perceived difficulties in functional ability than participant self-ratings relative to objective test scores (Martyr and Clare, 2018). Caregiver ratings have been associated with age of the person with dementia, neuropsychiatric symptoms, and/or cognitive ability, but can be related to caregiver stress (Martyr et al., 2012; Clare et al., 2012a; Martyr and Clare, 2018). Differences in kin relationship have also been found to affect ratings, with coresident spouses rating differently to non-coresident and non-spousal caregivers (Lin et al., 2017; Hackett et al., 2020), though this has not been consistently reported (Martyr et al., 2012; Alexander et al., 2021a; Martyr et al., 2022).

The effect of these factors on caregiver ratings may be due either to caregiver stress influencing the extent of difficulties reported as experienced by the person with dementia, or to presence of more difficulties in the person with dementia increasing caregiver stress. Caregiver burden is typically more closely associated with ratings of functional ability than with objective measures of functional ability (Razani et al., 2007), suggesting it is more likely that caregiver stress or burden influences perception of difficulties. More negative caregiver ratings in a range of domains have been associated with caregiver stress and/or burden (Clare et al., 2012a; Conde-Sala et al., 2013). This association may be more common in female caregivers and those with concomitant physical and/or mental health problems (Conde-Sala et al., 2013; Perales et al., 2016). Caregivers themselves differ in their beliefs or understanding about dementia (Quinn et al., 2019) which may reflect variation in the degree and/or nature of information provision (Dooley et al., 2015), or individual ways of handling information. This may influence caregivers’ evaluation of abilities in the person with dementia. It may be helpful in some cases to view and interpret the discrepancy between ratings as reflecting the level of actual performance rather than assuming the caregiver rating is more accurate than the corresponding rating by the person with dementia.

Few studies have explored wider relationship factors that might explain variation in caregiver ratings or the resultant discrepancy in views. It is unclear how positive aspects of a relationship relate to caregiver ratings or agreement in perspective. Better quality of relationship has been associated with more positive caregivers’ ratings and a smaller discrepancy in views regarding socioemotional functioning (Nelis et al., 2011). Lower levels of criticism and less emotional over-involvement within couples were associated with a smaller discrepancy in ratings of cognitive ability (Hanson and Clarke, 2013). For men adjusting to a diagnosis of dementia, the responses and coping processes of wives can mitigate the impact of the diagnosis and reduce the need for ‘more drastic self-reappraisal’ (Pearce et al., 2002). There does not appear to be an association between cognition and ratings of relationship quality, or the discrepancy between self- and informant ratings of relationship quality (Clare et al., 2012b), suggesting that having a good quality relationship is not dependent on the degree of cognitive difficulties experienced by the person with dementia. It would be interesting to see whether a closer relationship, with a shared outlook on life such as shared religious beliefs, helps foster a mutual understanding of the condition with less divergence of ratings. Alternatively, caregivers might rate less negatively to avoid appearing disloyal or critical of a partner who shows little awareness of difficulties.

Previously, awareness of having the condition of dementia was investigated (Alexander et al., 2021a,b) using a checklist which screens for awareness of difficulties from the Representations and Adjustment to Dementia Index (RADIX; Quinn et al., 2018). Awareness was considered low if none of the items describing difficulties were endorsed by the person with dementia. In the Improving the experience of Dementia and Enhancing Active Life programme (IDEAL; Clare et al., 2014), the checklist was also completed by the caregiver as informant, documenting perceived changes in the person with dementia. Some people with dementia may not experience all these difficulties, and therefore may accurately endorse fewer items on the nine-item checklist. However, some concordance is expected between the number of items endorsed by the person with dementia and the caregiver. As often used in awareness measures (Alexander et al., 2021b), a larger difference in the number of items endorsed might be considered altered awareness of difficulties by the person with dementia.

In this study, we investigated the caregiver RADIX checklist responses, and the difference between responses by the person with dementia (also referred to as the participant) and the caregiver. These exploratory analyses focused on co-resident spouse/partner dyads only. The study aims to answer these two research questions: 1. What characteristics of the person with dementia and the caregiver are associated with caregiver responses to the RADIX checklist? 2. To what extent can any differences in perspective between the person with dementia and the caregiver be explained in terms of dementia-related factors, and/or characteristics of the dyadic relationship?

## Materials and methods

### Study design

This is a cross-sectional study using baseline data from the IDEAL cohort, with information from dataset version 7.

Ethical approval for IDEAL was given by the Wales Research Ethics Committee 5 (reference 13/WA/0405) and the Ethics Committee of the School of Psychology, Bangor University (reference 2014–11684). IDEAL was registered with UKCRN, registration number 16593.

### Setting

Data were collected between 2014 and 2016 in 29 NHS research networks in England, Scotland, and Wales from community-dwelling people with mild-to-moderate dementia, interviewed at home.

### Participants

Inclusion criteria for IDEAL participants included Mini-Mental State Examination (MMSE; Folstein et al., 1975) score of 15 or above, confirmed diagnosis of dementia of any type made by clinicians at participating recruitment centers, and capacity to provide informed consent. There was no minimum age criterion specified. Full details of the inclusion and exclusion criteria can be found in the study protocol (Clare et al., 2014). Caregivers, defined as the primary person providing practical or emotional unpaid support, were recruited where possible, and acted as informants. There were 1,537 people with dementia at baseline. Of these, 1,038 had coresident spouse/partner caregivers who took part, and these dyads comprise the sample for this study.

### Measures

See Supplementary text for a detailed description for all measures.

### Awareness of condition

Taken from the Representations and Adjustment to Dementia Index (Quinn et al., 2018), the nine-item RADIX checklist records difficulties commonly experienced in dementia; participants and caregivers completed the self-rated and informant-rated versions, respectively. The total number of items endorsed was summed for the participant (Participant-RADIX) and for the caregiver (Caregiver-RADIX). RADIX-Difference was computed for each dyad by subtracting Participant-RADIX from Caregiver-RADIX. This can be considered as an index of awareness of difficulties and/or condition on the part of the person with dementia with larger positive differences indicating lower awareness.

### Other measures

#### Person with dementia

Cognition was assessed with the MMSE (Folstein et al., 1975). Mood was self-reported with the Geriatric Depression Scale-10 (GDS-10; Almeida and Almeida, 1999). Comorbidity was measured with the Charlson Comorbidity Index (CCI; Charlson et al., 1987, 2008), recording the number of health conditions other than dementia. Self-report items from the Positive Affect Index (PAI; Bengtson and Schrader, 1982; Clare et al., 2012b) were used to indicate current relationship quality.

#### Informant-reported

Functional ability was reported by the caregivers as informants on the modified Functional Activities Questionnaire (FAQ; Pfeffer et al., 1982; Martyr et al., 2012), with higher scores indicating greater perceived functional difficulties. The number of neuropsychiatric symptoms was reported using the Neuropsychiatric Inventory Questionnaire (NPI-Q; Kaufer et al., 2000; Morris and National Alzheimer's Coordinating Center, 2008).

#### Caregiver

Caregivers reported their mood with the Center for Epidemiologic Studies Depression Scale-Revised (CESD-R; Eaton et al., 2004). Stress associated with the caring role was reported with the Relative Stress Scale (RSS; Greene et al., 1982). Caregiver health was self-reported with the number of conditions on the CCI, and a self-rated single-item health question. Caregivers also completed questions from the PAI regarding current relationship quality.

### Demographic information

For the person with dementia, age, sex, and time since diagnosis were self-reported. Dementia type was recorded from medical records. For the caregiver, age, sex, education, and daily hours of caregiving were self-reported. For the dyad, area deprivation quintile was derived from nationally available deprivation indices and postcode information (Wu et al., 2018). Age difference was calculated (participant age minus caregiver age). The participant and caregiver were each asked for their religion, if any. Participant and caregiver individually rated personal importance of religion, allowing derivation of shared importance of religion for the dyad, which is a categorical variable based on their individual responses.

### Analyses

Descriptive statistics were reported for demographic details. RADIX total scores for participant and caregiver were used to calculate the RADIX-Difference. Caregiver-RADIX showed a left skew with a ceiling effect. To make the distribution more suitable for analysis, the Caregiver-RADIX scale was reversed in this regression alone, meaning that a lower score indicates more reported difficulties, and a negative binomial model was fitted. RADIX-Difference was investigated using exploratory univariable and multivariable linear regressions. Assumptions for independence of residuals, normality and homoscedasticity were met and independent variables were checked for multicollinearity. Participant and caregiver age group, participant sex, and dementia type were included as covariates in both regression models. Bonferroni correction for multiple comparison was applied to the analyses.

Missing data for the predictor variables were assumed to be missing at random; multiple imputation was used to generate 25 imputed datasets using the *mice* package in R. Estimates were combined according to Rubin’s rules (Rubin, 1996). Statistical analysis was conducted in IBM SPSS v28.0.

## Results

For 1,038 dyads with a coresident spouse/partner as caregiver, the mean age was 75.08 for participants and 72.43 for caregivers. Most dyads were heterosexual couples, apart from eight same-sex couples (one male, seven female). Nearly all the dyads were from white ethnic groups (99%), with over 96% from white British backgrounds. Few dyads lived in the most deprived areas (6.9%) while 32.4% lived in the least deprived areas. For dyads where importance of religion was shared, the reported religion by both members of the dyad was Christianity in 95% of cases. For further details see Supplementary Table 1. For sex differences between variables see Supplementary Table 2.

### RADIX responses

**Figure 1:**
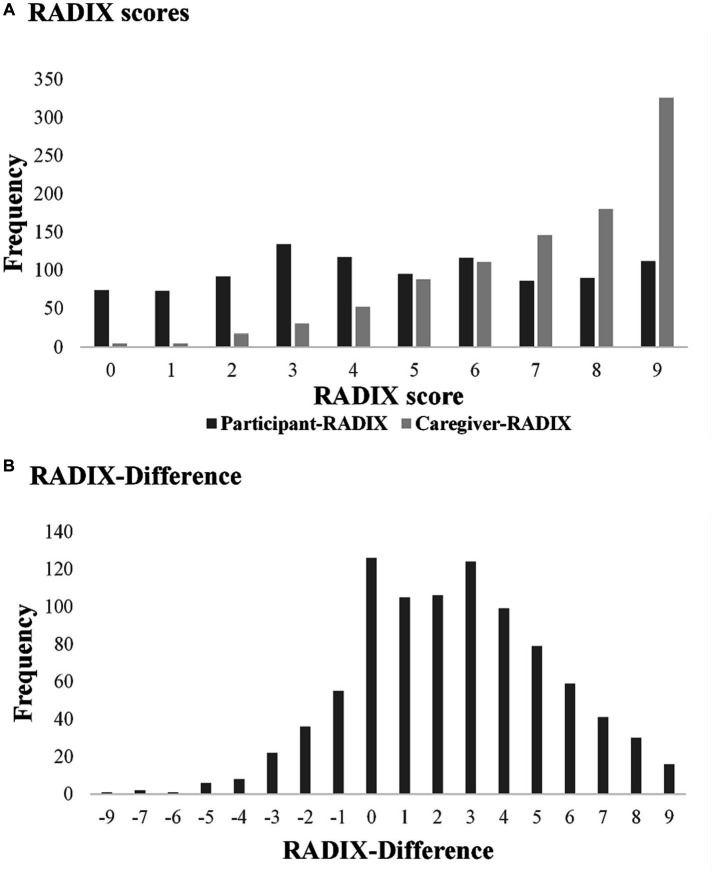
RADIX checklist scores, Participant-RADIX: Person with dementia RADIX checklist total score (*n* = 989). Caregiver-RADIX: Caregiver-informant RADIX checklist total score (*n* = 960). RADIX-Difference: Caregiver-RADIX minus Participant-RADIX (*n* = 916).

RADIX checklist data was complete for 989 participants and 960 caregivers, allowing calculation of the RADIX-Difference for 916 dyads. The RADIX total scores ranged from 0 to 9 for both participants and caregivers; however, the modal values were 3 for participants and 9 for caregivers (see Figure 1A). Within dyads, the difference between scores ranged from −9 to +9, with modal value of 0 (see Figure 1B). For 14% of dyads, the participant score was higher than the caregiver score, in most cases differing by one or two points only. For dyads where the RADIX-Difference was zero (n = 126), the participant and caregiver RADIX scores were generally high, typically endorsing 9/9 items; see Supplementary Figure 1.

### Caregiver-RADIX

Univariable analysis showed the following factors were associated with Caregiver-RADIX: participant age group, sex, and dementia type, MMSE, informant-rated NPI-Q and FAQ. Age difference, and caregiver factors of age group, daily hours of caregiving, PAI, CESD-R and RSS were also associated with Caregiver-RADIX. In multiple regression, participant age group, participant sex, dementia type, NPI-Q, FAQ, and RSS were associated with Caregiver-RADIX. The remaining variables did not contribute to the model. More perceived difficulties (i.e., lower reversed Caregiver-RADIX score) were associated with higher NPI-Q, FAQ, and RSS. More RADIX difficulties were reported for people with dementia with Lewy bodies, and fewer difficulties where dementia type was unspecified, compared to people with Alzheimer’s disease. For participants aged under 80, caregivers endorsed up to 2 more RADIX items (22%) compared to participants aged over 80. For male participants, caregivers (predominantly female) endorsed 1–2 more items (16%) than for female participants (with predominantly male caregivers); see Table 1. Caregivers of male participants reported significantly higher stress (mean RSS 20.82, SD 9.78) than caregivers of female participants (mean RSS 16.33, SD 8.96), although there was little difference in participants’ cognitive ability (males mean MMSE 23.43, SD 3.70; females mean MMSE 22.56, SD 3.56). There was a weak association between participant sex and daily hours of caregiving: Pearson χ_2_ (2) =7.797, *p* = 0.021; Cramer’s *V* = 0.087, with caregivers of male participants more likely to report spending 10+ daily hours of caregiving.

**Table 1:** Reversed caregiver-RADIX univariable and multivariable negative binomial regression.

	Univariable regression			Multivariable regression		
	Incidence rate ratio	(95% CI)	*p*-value	Incidence rate ratio	(95% CI)	*p*-value
*Participant variables*						
Participant age group						
<65	0.78	(0.59, 1.02)	0.066	0.80	(0.59, 1.09)	0.157
65–69	0.89	(0.71, 1.12)	0.328	0.83	(0.65, 1.05)	0.116
70–74	0.81	(0.66, 0.99)	**0.043**	0.78	(0.64, 0.96)	**0.017**
75–79	0.84	(0.70, 1.02)	0.078	0.87	(0.74, 1.01)	0.064
80+	Ref.			Ref.		
*Participant sex*						
Male	0.72	(0.62, 0.83)	<0.001*	0.84	(0.74, 0.96)	**0.007**
Female	Ref.			Ref.		
*Dementia type*						
AD	Ref.			Ref.		
VaD	0.78	(0.61, 1.00)	**0.048**	0.86	(0.71, 1.04)	0.119
Mixed AD/VaD	0.92	(0.76, 1.11)	0.381	0.91	(0.79,1.05)	0.193
FTD	0.69	(0.48, 1.01)	0.057	0.78	(0.58, 1.05)	0.100
PDD	0.79	(0.54, 1.17)	0.236	1.02	(0.76, 1.37)	0.907
DLB	0.37	(0.23, 0.59)	<0.001*	0.61	(0.40, 0.91)	**0.017**
Unspecified/other	1.13	(0.75, 1.71)	0.571	1.51	(1.12, 2.04)	**0.006**
*Time since diagnosis*						
<1 yr	Ref.					
1-2 yrs	0.86	(0.73, 1.01)	0.071			
3 + yrs	0.86	(0.69, 1.06)	0.159			
MMSE	1.06	(1.04, 1.08)	<0.001*	1.00	(0.99, 1.02)	0.812
*Informant-rated variables*						
FAQ	0.93	(0.93, 0.94)	<0.001*	0.95	(0.94, 0.96)	<0.001*
NPI-Q number of symptoms	0.83	(0.80, 0.85)	<0.001*	0.95	(0.92, 0.98)	<0.001*
*Caregiver variables*						
Caregiver age group						
<65	0.70	(0.55, 0.89)	**0.003**	0.94	(0.70, 1.26)	0.680
65–69	0.74	(0.59, 0.93)	**0.01**	0.98	(0.77, 1.23)	0.836
70–74	0.74	(0.60, 0.91)	**0.005**	0.91	(0.75, 1.11)	0.362
75–79	0.87	(0.70, 1.08)	0.212	1.00	(0.84, 1.18)	0.996
80+	Ref.			Ref		
Age difference	0.98	(0.96, 0.99)	<0.001*			
*Area deprivation*						
Q1 Most deprived	0.90	(0.67, 1.21)	0.491			
Q2	0.83	(0.66, 1.05)	0.127			
Q3	0.95	(0.78, 1.15)	0.587			
Q4	0.85	(0.71, 1.03)	0.100			
Q5 Least deprived	Ref.					
*Caregiver education*						
No qualification	1.02	(0.84, 1.24)	0.835			
School leaving cert. Age 16	0.85	(0.69, 1.04)	0.112			
School leaving cert. Age 18	Ref.					
University	0.96	(0.79, 1.17)	0.698			
RSS	0.95	(0.94, 0.95)	<0.001*	0.98	(0.97, 0.99)	<0.001*
CESD-R	0.96	(0.95, 0.97)	<0.001*	1.00	(0.99, 1.01)	0.927
Caregiver CCI no. of conditions	0.96	(0.91, 1.01)	0.149			
Caregiver PAI current	1.07	(1.05, 1.09)	<0.001*	1.00	(0.99, 1.02)	0.771
*Caregiver self-rated health*						
Poor/Very poor	0.85	(0.63, 1.14)	0.271			
Fair	0.82	(0.67, 0.99)	**0.035**			
Good	Ref.					
Very good	0.96	(0.80, 1.16)	0.673			
Excellent	1.13	(0.86, 1.48)	0.392			
<1 h	2.40	(2.02, 2.85)	<0.001*	0.99	(0.85, 1.16)	0.901
1-10 h	1.21	(1.04, 1.42)	**0.016**	0.95	(0.83, 1.08)	0.444
10 + h	Ref.			Ref.		

Bold font indicates significant *p*-level < 0.05. *Remains significant after correction for multiple comparisons.

AD, Alzheimer’s disease; VaD, Vascular dementia; FTD, Frontotemporal dementia; PDD, Parkinson’s disease dementia; DLB, Dementia with Lewy bodies; MMSE, Mini-Mental State Examination; FAQ, Functional Activities Questionnaire; NPI-Q, Neuropsychiatric Inventory Questionnaire; RSS, Relative Stress Scale, CESD-R, Center for Epidemiologic Studies Depression Scale-Revised; CCI, Charlson Comorbidity Index; PAI, Positive Affect Index.

### RADIX-difference

Univariable regression showed that the participant factors age group, dementia type, MMSE, GDS-10, CCI, FAQ and NPI-Q were associated with the RADIX-Difference. Also associated were caregiver variables of age group, RSS and PAI. In multivariable regression, participant sex, dementia type, FAQ, GDS-10, participant CCI and caregiver RSS were associated with RADIX-Difference, with a non-significant trend showing for participant age group. The remaining variables did not contribute to the model. In younger participant age groups compared to participants aged over 80, and in participants with more depressed mood, smaller RADIX-Differences were seen suggesting greater concordance between participant and caregiver. Higher participant CCI score was also associated with smaller RADIX-Difference. Greater RADIX-Difference, suggesting lower concordance between participant and caregiver, was associated with higher FAQ and RSS; see Table 2.

**Table 2:** RADIX-difference univariable and multivariable regression.

	Univariable regression			Multivariable regression*		
Variables	B unstandardized	(95.0% CI)	*p*-value	B unstandardized	(95.0% Cl)	*p*-value
*Participant variables*						
Participant age group						
<65	−1.38	(−2.10, −0.66)	**<0.001** * *	−0.84	(−1.79, 0.12)	0.088
65–69	−0.79	(−1.40, −0.19)	**0.01**	−0.21	(−0.94, 0.53)	0.583
70–74	−0.50	(−1.06, 0.05)	0.073	0.07	(−0.56, 0.70)	0.832
75–79	−0.06	(−0.57, 0.44)	0.807	0.37	(−0.13, 0.87)	0.148
80+	Ref.			Ref.		
*Participant sex*						
Male	0.32	(−0.09, 0.73)	0.125	0.20	(−0.22, 0.61)	0.360
Female	Ref.			Ref.		
*Dementia type*						
AD	Ref.			Ref.		
VaD	−0.30	(−0.95, 0.34)	0.358	−0.05	(−0.64, 0.54)	0.873
Mixed AD/VaD	0.39	(−0.12, 0.90)	0.129	0.41	(−0.06, 0.87)	0.086
FTD	0.38	(−0.61, 1.36)	0.454	0.44	(−0.46, 1.33)	0.341
PDD	−1.15	(−2.16, −0.14)	**0.026**	−0.80	(−1.71, 0.12)	0.089
DLB	−0.83	(−1.84, 0.18)	0.107	−1.18	(−2.10, −0.26)	**0.012**
Unspecified/Other	−0.70	(−1.89, 0.49)	0.246	−0.91	(−1.97, 0.14)	0.090
MMSE	−0.10	(−0.16, −0.05)	**<0.001** * *	0.00	(−0.05, 0.05)	0.942
GDS-10	−0.37	(−0.45, −0.29)	**<0.001** * *	−0.39	(−0.47, −0.31)	**<0.001** * *
Participant PAI current	0.02	(−0.04, 0.07)	0.516			
Participant CCI no. of non-dementia conditions	−0.28	(−0.40, −0.16)	**<0.001** * *	−0.18	(−0.30, −0.05)	**0.005**
*Informant-rated variables*						
FAQ	0.11	(0.08, 0.13)	**<0.001** * *	0.10	(0.07, 0.13)	**<0.001** * *
NPI-Q number of symptoms	0.16	(0.08, 0.23)	**<0.001** * *	0.02	(−0.07, 0.11)	0.657
*Caregiver variables*						
Caregiver age group						
<65	−0.43	(−1.05, 0.18)	0.170	0.13	(−0.58, 0.84)	0.728
65–69	0.37	(−0.21, 0.94)	0.212	0.15	(−0.40, 0.69)	0.597
70–74	Ref.			Ref.		
75–79	0.43	(−0.14, 0.99)	0.141	0.22	(−0.33, 0.77)	0.430
80+	0.78	(0.20, 1.36)	**0.008**	0.69	(0.05, 1.33)	**0.034**
RSS	0.06	(0.04, 0.08)	**<0.001** * *	0.03	(0.00, 0.05)	**0.025**
CESD-R	0.00	(−0.03, 0.03)	0.934			
Caregiver PAI current	−0.08	(−0.12, −0.04)	**<0.001** * *	−0.03	(−0.07, 0.01)	0.188
Caregiver CCI no. of conditions	0.06	(−0.07, 0.20)	0.372			
*Dyadic variables*						
Area deprivation						
Q1 Most deprived	−0.45	(−1.23, 0.33)	0.26			
Q2	−0.41	(−1.03, 0.22)	0.202			
Q3	−0.05	(−0.58, 0.49)	0.863			
Q4	0.01	(−0.50, 0.52)	0.967			
Q5 Least deprived	Ref.					
Age difference	0.03	(−0.00, 0.07)	0.07			
*Shared importance of religion*						
Not important to both	0.12	(−0.43, 0.66)	0.68			
Important to participant only	−0.09	(−0.64, 0.46)	0.751			
Important to caregiver only	0.48	(−0.13, 1.08)	0.124			
Important to both	Ref.					

* *R*-square = 0.252, adjusted *R*-square = 0.234, *F*(22, 893) = 13.67, *p* < 0.001.

Bold font indicates significant at level *p* < 0.05. **Remains significant after correction for multiple comparison.

AD, Alzheimer’s disease; VaD, Vascular dementia; FTD, Frontotemporal dementia; PDD, Parkinson’s disease dementia; DLB, Dementia with Lewy bodies; MMSE, Mini-Mental State Examination; GDS-10, Geriatric Depression Scale-10; PAI, Positive Affect Index; CCI, Charlson Comorbidity Index; FAQ, Functional Activities Questionnaire; NPI-Q, Neuropsychiatric Inventory Questionnaire; RSS, Relative Stress Scale; CESD-R, Center for Epidemiologic Studies Depression Scale-Revised.

## Discussion

In a large sample of people with mild-to-moderate dementia, the RADIX checklist was used to examine reported difficulties by the caregiver acting as informant and the person with dementia, taking the difference between the ratings as an index of awareness of condition in the person with dementia. In this exploratory analysis caregiver-RADIX was associated with caregiver ratings of functional ability and number of neuropsychiatric symptoms in the person with dementia, suggesting some consistency in how informant ratings were made. For younger people with dementia and those with dementia with Lewy bodies, caregivers reported more difficulties, unrelated to cognitive score. There was little evidence of caregiver characteristics or the dyadic relationship affecting caregiver ratings or the resulting RADIX-Difference. However, female caregivers reported more difficulties in the person with dementia than male caregivers. Higher levels of caregiver stress were associated with reporting of more RADIX difficulties, and both were higher for female caregivers. Generally, caregivers noticed more difficulties than people with dementia, and the discrepancy score for condition, taken as an indication of awareness, was also associated with higher caregiver stress. Lower awareness was seen in older participant age groups and those with poorer perceived functional ability, and higher awareness in more depressed participants. Dementia type and participant comorbidity had some effect, with more awareness seen in people with dementia with Lewy bodies, and with greater comorbidity.

Fewer difficulties were reported by caregivers for participants over 80. This contrasts with findings in a smaller study, where informants rated functional ability and memory as more impaired in older participants (Martyr et al., 2012; Clare et al., 2012a). However, the larger RADIX-Difference suggesting lower awareness in older age groups was consistent with other studies (Clare et al., 2012a; Alexander et al., 2021a; Martyr et al., 2022). The contrasting findings could be attributed to those measures requiring different judgments and facilitating greater objectivity for caregivers. Caregivers of people with dementia over 80 may have lower expectations or more generous inclinations in RADIX ratings, or perhaps the general difficulties are less remarkable in the context of other age-related problems. Perceived functional ability, but not the objective cognitive score, was associated with Caregiver-RADIX and RADIX-Difference. This reflects previous work describing the limitations of cognitive scores to fully explain variations in awareness at any stage of dementia (Clare, 2004; Clare et al., 2012b; Sunderaraman and Cosentino, 2017).

We found a sex difference in Caregiver-RADIX with female caregivers more likely to report more difficulties; a finding not explained by levels of participant cognition and with only marginal sex difference in daily hours of caregiving. Female caregivers also reported more stress than male caregivers unexplained by hours of caregiving. However, the RADIX-Difference findings suggest caregiver stress is associated with a larger discrepancy score, with no sex difference. Sex differences in managing the caregiver role have been described, with differences in the expectations and the impact of caregiving (Morris et al., 1991). Females may experience a higher subjective burden of care (Quinn et al., 2012; Brodaty et al., 2014) and more emotional involvement, with male caregivers taking a more task-orientated approach (Morris et al., 1991). In a study that included other family caregivers, caregiver ratings of functional and cognitive ability were more strongly associated with caregiver burden than sex of the caregiver, but female caregivers, particularly daughters, gave more negative ratings (Conde-Sala et al., 2013).

Consistent with our findings, caregiver stress has been associated with lower awareness in the person with dementia when assessed with discrepancy measures reliant on caregiver ratings (Martyr et al., 2012; Clare et al., 2012a; Mayelle et al., 2022), or with a clinician rating of global awareness (Turró-Garriga et al., 2013). It seems likely that caring for someone who shows little acknowledgement of their difficulties is inherently stressful. However, when awareness was categorized using the participant self-reported RADIX checklist alone (Alexander et al., 2021a), no link was seen with caregiver stress. A potential negative bias in reporting difficulties by caregivers who are stressed, particularly females, cannot be excluded, and the study does not distinguish between these possibilities.

Poorer relationship quality has been associated with more adverse ratings of socioemotional functioning by caregivers (Clare et al., 2012a), and in the context of more carer stress in spousal dyads (Clare et al., 2012b). Female caregivers have reported lower relationship quality than male caregivers (Quinn et al., 2012). However, neither Caregiver-RADIX nor RADIX-Difference were associated with current relationship quality in our study, where nearly all the dyads were heterosexual spousal couples. A possible explanation for the sex difference in caregiver ratings and stress might be that caring for a man/husband with dementia is more stressful than caring for a woman/wife with dementia. In this cohort of older couples, traditional marriage roles, perhaps of protector and provider, may influence how a male caregiver provides care for his wife/partner with dementia (Morris et al., 1991), but also how a man with dementia receives care from his female spouse/partner. In male care-recipients, reluctance to accept assistance beyond the customary household chores could feasibly create more stress for the caregiver and enhance the caregiver’s perception of difficulties. Female caregivers may also find it harder to help a male person with dementia to wash, dress, transfer themselves, and other more physically demanding tasks. This is unlikely to be applicable in the present study as people were primarily in the mild-to-moderate stages of dementia and few had problems with more basic activities of daily living (Martyr et al., 2022).

We looked at the shared importance of religion to investigate whether this represented a shared outlook and promoted closer perspectives within the dyad regarding difficulties experienced. For individuals with religious beliefs, maintaining faith practices can be an important part of maintaining self-identity despite dementia, providing a sense of continuity and stability as well as involvement in a community (Daly et al., 2019). This can support coping strategies that normalize experiences and enable maintenance of ‘life as usual’ (Bjørkløf et al., 2019), and could affect expressed awareness of condition. For couples living with dementia together, maintaining the dyadic identity can be important for the quality of relationship (Colquhoun et al., 2019). We speculated that couples with a shared importance of religion might have more similar views of the difficulties experienced, but no association was shown. A more nuanced investigation might consider more detailed information about past and ongoing religious activity and perspectives within the dyad.

Consistent with previous research using participant-reported difficulties only (Alexander et al., 2021a), and discrepancy awareness measures in other domains (Clare et al., 2012a; Lacerda et al., 2020; Martyr et al., 2022), smaller RADIX-Difference was related to more depressed participant mood. Whether low mood is a cause or consequence of higher awareness of difficulties remains difficult to determine (Azocar et al., 2021). Negative bias in self-perception due to depression is known to exist (Mograbi and Morris, 2014; Martyr et al., 2019), and in a non-dementia population, participants with depressed mood reported more difficulties in functioning than their informants (Verrijp et al., 2022). Reflecting findings about awareness of functional difficulties and comorbidity in the IDEAL cohort (Martyr et al., 2022), greater participant comorbidity was associated with smaller RADIX-Difference score. However, with a small effect size, this may only be meaningful for people with a very high number of comorbid conditions. Having multiple health problems, including depression, could plausibly make everyday difficulties more noticeable, and/or prompt more feedback from others, leading to more acceptance and acknowledgement of condition.

A small minority of caregivers reported fewer difficulties than their spouse/partner with dementia, or even none. This is likely to reflect an underestimation by the caregiver, as difficulties with everyday activities must be present for a diagnosis of dementia to be made (World Health Organization, 2019/2021; American Psychiatric Association, 2022). However, it could also reflect an exaggerated view of difficulties on the part of the person with dementia. Other studies have revealed heightened awareness of difficulties, where people with dementia appear to underestimate their ability compared to caregivers’ ratings (Marková et al., 2014; Martyr et al., 2022). This too could be indicative of poor awareness about the degree of difficulties, although when compared with objective performance caregivers generally overestimate difficulties (Martyr and Clare, 2018; Camino et al., 2022). For most dyads where RADIX-Difference was zero, a high number of difficulties were reported by both members, i.e., there was agreement that difficulties were substantial. The possibility of bias in reporting by stressed caregivers, or by people with dementia who are depressed has been discussed above. Thus, the drawbacks of discrepancy measures to assess awareness are recognized. However, caregivers are uniquely placed to provide valuable observations, and the demonstration of a difference in perspective has additional utility. The ratings by the caregiver and the person with dementia provide information about their experience at that time and highlight areas of agreement or disagreement. This information could be used to identify where support is needed. In clinical practice, variations in awareness affect people with dementia (Alexander et al., 2021a), and raise real concerns for caregivers (Turró-Garriga et al., 2016) and healthcare professionals (Dooley et al., 2015), relevant for communication and care-planning. If using caregiver ratings of difficulties to benchmark awareness, clinicians should recognize the range of possible reasons for differences in perspective. Clinicians require objective information about the difficulties experienced by the person with dementia. Information from caregivers and people with dementia should be interpreted in the context of the dyad and emotional responses to difficulties experienced. Caregivers could be offered education about how to respond to situations if their ratings suggest low appreciation of difficulties. This might include dyadic support to negotiate a shared understanding of difficulties, with practical solutions, and separate psychological support for the caregiver and the person with dementia. Education for clinicians and social care providers is needed to increase the understanding about awareness in dementia, adding to the provision of tailored support.

### Strengths and limitations of the study

The study makes novel use of the validated RADIX screening checklist as a discrepancy measure for awareness of condition. It investigates a large sample of people recruited from memory clinics in Great Britain. The sample comprised spouse/partner caregiver dyads reducing potential variability arising from inclusion of other kin relationships. The study adds research on awareness of condition, and exploration of informant ratings. However, diversity of ethnic group and sexuality was low, and findings may not be generalizable to other groups, or to caregiver relationships other than coresident spouse/partners. Effect sizes and variance explained are small. Awareness in dementia is complex (Clare et al., 2011), likely to be influenced by multiple factors (Clare et al., 2012a; Martyr et al., 2022; Alexander et al., 2022a,b), and manifest in a range of individual profiles (Mayelle et al., 2022). Consequently, the main limitation is the reliance on ratings to assess awareness of condition. As discussed above, ratings are subject to numerous influences and biases. Combining ratings with more objective assessments made by clinicians would likely mitigate this limitation. Future research could include these nine checklist items, or a short select list of items, in a memory clinic setting. This could help delineate whether ratings made by the caregiver or person with dementia are more consistent with objective data regarding presence of these nine specific difficulties. There is scope to further explore psychosocial influences on informant ratings and awareness, by investigating other aspects of the dyadic relationship.

## Conclusion

When caregivers report on difficulties noticed in spouses/partners with dementia, difficulties may be under-reported for older age groups. Female caregivers may perceive more difficulties than male caregivers. Negative caregiver ratings and larger differences in perspective may indicate higher caregiver stress. However, there is little evidence that other characteristics of the caregiver and dyadic relationship influence informant ratings or the difference in perspective. Cognitive scores have limited value for understanding caregivers’ perspective or gauging awareness of condition. Comorbidities, including depression, might enhance personal awareness of everyday difficulties. Accuracy of informant ratings cannot be assumed but, along with the difference in perspective about difficulties, these lend valuable information about the experience of the caregiver and the person with dementia and could be used to direct tailored support for the dyad.

## Data availability statement

Publicly available datasets were analyzed in this study. IDEAL data were deposited with the UK data archive in April 2020. Details of how to access the data can be found here: https://reshare.ukdataservice.ac.uk/854293/.

## Ethics statement

The studies involving humans were approved by the Wales Research Ethics Committee 5 (reference 13/WA/0405) and the Ethics Committee of the School of Psychology, Bangor University (reference 2014–11,684). The studies were conducted in accordance with the local legislation and institutional requirements. The participants provided their written informed consent to participate in this study.

## Author contributions

CA: Conceptualization, Formal analysis, Investigation, Writing – original draft. AM: Data curation, Writing – review & editing, Funding acquisition. LG: Data curation, Formal analysis, Software, Writing – review & editing. CQ: Writing – review & editing, Funding acquisition. CP: Writing – review & editing. RM: Writing – review & editing, Funding acquisition. LC: Conceptualization, Writing – review & editing, Funding acquisition.
