# Supplementary material for: Dyadic influences on awareness of condition in people with dementia: findings from the IDEAL cohort
Source: Front Aging Neurosci. 2023 Dec 11;15:1277336. doi: 10.3389/fnagi.2023.1277336 (PMC10749333; doi:10.3389/fnagi.2023.1277336)
Supplement: Supplementary file 1 [file Data_Sheet_1.docx]

**SUPPLEMENTARY MATERIAL**

**Supplementary Text**

**Measure description**

***Awareness of condition***

Taken from the Representations and Adjustment to Dementia Index (RADIX; Quinn et al., 2018; Quinn et al., 2019) the nine-item RADIX checklist was self-rated by people with dementia (the participants), and informant-rated by caregivers. The checklist was designed to screen for awareness of difficulties commonly experienced in dementia, and the participant self-report has previously been used to categorize awareness of condition (Alexander et al., 2021; Alexander et al., 2022). In this study, the participant and informant responses were compared to assess the difference or discrepancy in perspective, which can be considered an index of awareness of difficulties or condition by the person with dementia. The total number of items endorsed was summed for the participant (Participant-RADIX) and for the caregiver as informant (Caregiver-RADIX). The difference between the number of items endorsed was calculated (Caregiver-RADIX minus Participant-RADIX) to find the RADIX-Difference.

Higher scores for Participant-RADIX and Caregiver-RADIX indicate more difficulties were reported. However, for the Caregiver-RADIX regression analyses alone, reversed scores were used i.e., lower scores indicated more difficulties reported.

For the RADIX-Difference, positive scores indicate that the person with dementia underestimated their difficulties compared to the informant, while negative scores indicate that the informant underestimated difficulties compared to the person with dementia. Larger scores represent a greater difference in perspective.

***Other measures***

**Person with dementia**: Cognition was assessed with the Mini-Mental State Examination (MMSE; Folstein et al., 1975). Scores range from 0 to 30, with higher scores indicating better cognitive ability. Scores of 10 or above are considered to indicate mild-to-moderate dementia. Mood was self-reported with the Geriatric Depression Scale-10 (GDS-10; Almeida and Almeida, 1999). This is a 10-item screening test for depression with possible scores from 0-10. Scores 4 and above are considered to indicate depressed mood. Comorbidity was measured with the Charlson Comorbidity Index (CCI; Charlson et al., 1987; Charlson et al., 2008) using the 2008 criteria, recording the number of conditions other than dementia. This was administered as a joint interview between the person with dementia and his/her caregiver. Total scores ranged between 0 and 22 (as dementia was not included in the score), with higher scores indicating more comorbidities. To assess quality of relationship, items taken from the optional element of the Positive Affect Index (PAI: Bengtson, 1982) were adapted for older people. Items were completed by the participant and used to indicate current quality of relationship (Clare et al., 2012). Each item was rated on a 6-point scale, with possible scores ranging from 5-30. Higher scores indicate better quality of relationship.

**Informant-reported**: Functional ability was reported by the caregiver as informant on the modified 11-item Functional Activities Questionnaire (FAQ; Pfeffer et al., 1982). This comprises items about instrumental activities of daily living such as shopping, preparing a meal, and managing financial records etc.; an additional item has been added concerning using the telephone (Martyr et al., 2012). Possible scores range from 0-33 with higher scores indicating greater perceived functional difficulties. The number of neuropsychiatric symptoms was reported using the Neuropsychiatric Inventory Questionnaire (NPI-Q; Kaufer et al., 2000) with wording taken from the National Alzheimer’s Coordinating Center version (Morris and National Alzheimer's Coordinating Center, 2008). Items screen for the presence of symptoms in 12 neuropsychiatric domains. Possible scores range from 0-12, with higher scores indicating more neuropsychiatric symptoms.

**Caregiver:** Caregivers reported their mood with the Center for Epidemiologic Studies Depression Scale-Revised (Eaton et al., 2004). This is a 20-item scale to measure symptoms of depression. Scores range from 0-60 with higher scores indicating greater depressive symptoms; scores of 16 or above are considered to indicate depressed mood. Stress associated with the caring role, which includes social upset, was reported with the Relative Stress Scale (RSS; Greene et al., 1982). This is a 15-item measure, with each item self-reported on a 5-point scale. Possible scores range from 0-60 with higher scores indicating greater stress. Caregiver health was self-reported with the Charlson Comorbidity Index, using the version as described above recording the number of conditions but including dementia, with scores ranging from 0 to 23. Self-rated health was also assessed using the question: “Overall, how would you rate your health in the past four weeks?” categorized very poor, poor, fair, good, very good, excellent. As numbers in the categories poor and very poor were small, these groups were collapsed into a single group poor/very poor for the analyses. Caregivers also completed questions from the Positive Affect Index (see above), regarding current relationship.

***Demographic information***

**Person with dementia:** Age was categorized into five groups, <65, 65-69, 70-74, 75-79, 80+. Time since diagnosis was categorized <1 year, 1-2 years, 3+ years. Dementia type was recorded as Alzheimer’s disease, vascular dementia, mixed Alzheimer’s disease and vascular dementia, frontotemporal dementia, dementia with Lewy bodies, Parkinson’s disease dementia, unspecified/other dementia.

**Caregiver:** Age was categorized into five groups, <65, 65-69, 70-74, 75-79, 80+. Education was categorized into no qualifications, school leaving certificate at 16 years, school leaving certificate at 18 years, university. Daily hours of caregiving were categorized into <1 hour, 1-10 hours, 10+ hours.

**Dyad:** Area deprivation quintile was derived from nationally available deprivation indices and postcode information (Wu et al., 2018), with the first quintile representing the most deprived areas, and the fifth quintile representing the least deprived areas. The person with dementia and the caregiver were individually asked for their religion, if any. Importance of religion was self-rated by the person with dementia and the caregiver, using a 7-point scale from not important to extremely important. The responses were recategorized to create new individual variables for importance of religion rated as important or not important. This allowed derivation of a dyadic variable for shared importance of religion with 4 categories: not important to both, important to both, important to participant only, important to caregiver only.

**References for Supplementary Text:**

Alexander, C.M., Martyr, A., Clare, L., and IDEAL Programme Research Team (2022). Changes in awareness of condition in people with mild‐to‐moderate dementia: longitudinal findings from the IDEAL cohort. *Int. J. Geriatr. Psychiatry* 37(4)**,** 4. doi: 10.1002/gps.5702.

Alexander, C.M., Martyr, A., Gamble, L.D., Savage, S.A., Quinn, C., Morris, R.G., et al. (2021). Does awareness of condition help people with mild-to-moderate dementia to live well? Findings from the IDEAL programme. *BMC Geriatr.* 21(1)**,** 511. doi: 10.1186/s12877-021-02468-4.

Almeida, O.P., and Almeida, S.A. (1999). Short versions of the Geriatric Depression Scale: a study of their validity for the diagnosis of a major depressive episode according to ICD‐10 and DSM‐IV. *Int. J. Geriatr. Psychiatry* 14(10)**,** 858-865. doi: 10.1002/(SICI)1099-1166(199910)14:10%3C858::AID-GPS35%3E3.0.CO;2-8.

Bengtson, V.L., Schrader S.S (1982). "Parent-child relations," in *Research Instruments in Social Gerontology: Social roles and social participation* ed. D.J. Mangon, Peterson, W.A.: University of Minnesota Press), 115-186.

Charlson, M.E., Charlson, R.E., Peterson, J.C., Marinopoulos, S.S., Briggs, W.M., and Hollenberg, J.P. (2008). The Charlson Comorbidity Index is adapted to predict costs of chronic disease in primary care patients. *J. Clin. Epidemiol.* 61(12)**,** 1234-1240. doi: 10.1016/j.jclinepi.2008.01.006.

Charlson, M.E., Pompei, P., Ales, K.L., and MacKenzie, C.R. (1987). A new method of classifying prognostic comorbidity in longitudinal studies: development and validation. *J. Chronic Dis.* 40(5)**,** 373-383. doi: 10.1016/0021-9681(87)90171-8.

Clare, L., Nelis, S.M., Whitaker, C.J., Martyr, A., Marková, I.S., Roth, I., et al. (2012). Marital relationship quality in early-stage dementia: perspectives from people with dementia and their spouses. *Alzheimer Dis. Assoc. Disord.* 26(2)**,** 148-158. doi: 10.1097/WAD.0b013e318221ba23.

Eaton, W.W., Muntaner, C., Smith, C., Tien, A., and Ybarra, M. (2004). "Center for Epidemiologic Studies Depression Scale: review and revision (CESD and CESD-R)," in *The use of psychological testing for treatment planning and outcomes assessment,* ed. M.E. Maruish. 3rd ed (Lawrence Erlbaum), 363-377.

Folstein, M.F., Folstein, S.E., and McHugh, P.R. (1975). “Mini-mental state”: a practical method for grading the cognitive state of patients for the clinician. *J. Psychiatr. Res.* 12(3)**,** 189-198. doi: 10.1016/0022-3956(75)90026-6.

Greene, J.G., Smith, R., Gardiner, M., and Timbury, G.C. (1982). Measuring behavioural disturbance of elderly demented patients in the community and its effects on relatives: a factor analytic study. *Age Ageing* 11(2)**,** 121-126. doi: 10.1093/ageing/11.2.121.

Kaufer, D.I., Cummings, J.L., Ketchel, P., Smith, V., MacMillan, A., Shelley, T., et al. (2000). Validation of the NPI-Q, a brief clinical form of the Neuropsychiatric Inventory. *J. Neuropsychiatry Clin. Neurosci.* 12(2)**,** 233-239. doi: 10.1176/jnp.12.2.233.

Martyr, A., Clare, L., Nelis, S.M., Marková, I.S., Roth, I., Woods, R.T., et al. (2012). Verbal fluency and awareness of functional deficits in early-stage dementia. *Clin. Neuropsychol.* 26(3)**,** 501-519. doi: 10.1080/13854046.2012.665482.

Morris, J.C., and National Alzheimer's Coordinating Center (2008). *NACC Uniform Data Set (UDS) Coding Guidebook for Initial Visit Packet.*

Pfeffer, R.I., Kurosaki, T.T., Harrah, C.H., Jr., Chance, J.M., and Filos, S. (1982). Measurement of functional activities in older adults in the community. *J. Gerontol.* 37(3)**,** 323-329. doi: 10.1093/geronj/37.3.323.

Quinn, C., Jones, I.R., Martyr, A., Nelis, S.M., Morris, R.G., and Clare, L. (2019). Caregivers’ beliefs about dementia: findings from the IDEAL study. *Psychol. Health* 34(10)**,** 1214-1230. doi: 10.1080/08870446.2019.1597098.

Quinn, C., Morris, R.G., and Clare, L. (2018). Beliefs about dementia: development and validation of the Representations and Adjustment to Dementia Index (RADIX). *Am. J. Geriatr. Psychiatry* 26(6)**,** 680-689. doi: 10.1016/j.jagp.2018.02.004.

Wu, Y.T., Clare, L., Jones, I.R., Martyr, A., Nelis, S.M., Quinn, C., et al. (2018). Inequalities in living well with dementia—The impact of deprivation on well‐being, quality of life and life satisfaction: results from the Improving the experience of Dementia and Enhancing Active Life study. *Int. J. Geriatr. Psychiatry* 33(12)**,** 1736-1742. doi: 10.1002/gps.4998.

**Supplementary Table 1.** Characteristics of participants and coresident spouse/partner caregivers (n=1038)

|  | **Participant** | | **Caregiver** | | **Dyad** | |
| --- | --- | --- | --- | --- | --- | --- |
| **Category** | **n** | **%** | **n** | **%** | **n** | **%** |
| Age group |  |  |  |  |  |  |
| <65 | 94 | 9.1 | 156 | 15.0 |  |  |
| 65-69 | 146 | 14.1 | 198 | 19.1 |  |  |
| 70-74 | 205 | 19.7 | 258 | 24.9 |  |  |
| 75-79 | 265 | 25.5 | 216 | 20.8 |  |  |
| 80+ | 328 | 31.6 | 210 | 20.2 |  |  |
| Sex |  |  |  |  |  |  |
| Male | 685 | 66.0 | 347 | 33.4 |  |  |
| Female | 353 | 34.0 | 691 | 66.6 |  |  |
| Dementia type |  |  |  |  |  |  |
| AD | 582 | 56.1 |  |  |  |  |
| VaD | 107 | 10.3 |  |  |  |  |
| Mixed AD/VaD | 199 | 19.2 |  |  |  |  |
| FTD | 43 | 4.1 |  |  |  |  |
| PDD | 39 | 3.8 |  |  |  |  |
| DLB | 39 | 3.8 |  |  |  |  |
| Unspecified/Other | 29 | 2.8 |  |  |  |  |
| Time since diagnosis |  |  |  |  |  |  |
| <1 year | 509 | 49.0 |  |  |  |  |
| 1-2 years | 313 | 30.2 |  |  |  |  |
| 3+ years | 138 | 13.3 |  |  |  |  |
| Missing | 78 | 7.5 |  |  |  |  |
| Area deprivation quintiles |  |  |  |  |  |  |
| Q1 Most deprived |  |  |  |  | 72 | 6.9 |
| Q2 |  |  |  |  | 139 | 13.4 |
| Q3 |  |  |  |  | 223 | 21.5 |
| Q4 |  |  |  |  | 267 | 25.7 |
| Q5 Least deprived |  |  |  |  | 336 | 32.4 |
| Missing |  |  |  |  | 1 | .1 |
| Education |  |  |  |  |  |  |
| No qualifications |  |  | 255 | 24.6 |  |  |
| School leaving certificate at age 16 |  |  | 233 | 22.4 |  |  |
| School leaving certificate at age 18 |  |  | 309 | 29.8 |  |  |
| University |  |  | 237 | 22.8 |  |  |
| Missing |  |  | 4 | .4 |  |  |
| Self-rated health |  |  |  |  |  |  |
| Poor/Very poor |  |  | 72 | 7.0 |  |  |
| Fair |  |  | 246 | 23.7 |  |  |
| Good |  |  | 373 | 35.9 |  |  |
| Very good |  |  | 249 | 24.0 |  |  |
| Excellent |  |  | 84 | 8.1 |  |  |
| Missing |  |  | 14 | 1.3 |  |  |
| Hours of caregiving per day |  |  |  |  |  |  |
| <1 hour |  |  | 206 | 19.8 |  |  |
| 1-10 hours |  |  | 368 | 35.5 |  |  |
| 10+ hours |  |  | 450 | 43.4 |  |  |
| Missing |  |  | 14 | 1.3 |  |  |
| Religion |  |  |  |  |  |  |
| No religion | 125 | 13.6 | 166 | 18.1 |  |  |
| Christian | 751 | 82.0 | 723 | 78.9 |  |  |
| Other* | 35 | 3.8 | 21 | 2.3 |  |  |
| Missing | 5 | 0.5 | 6 | 0.7 |  |  |
| Shared importance of religion |  |  |  |  |  |  |
| Not important to both |  |  |  |  | 160 | 15.4 |
| Important to both |  |  |  |  | 574 | 55.3 |
| Important to participant only |  |  |  |  | 163 | 15.7 |
| Important to caregiver only |  |  |  |  | 127 | 12.2 |
| Missing |  |  |  |  | 14 | 1.3 |
|  | **N Valid** | **Mean** | **SD** | **Range** | **Missing (%)** |  |
| MMSE | 1037 | 23.13 | 3.68 | 14 to 30 | 1 (0.1) |  |
| GDS-10 | 937 | 2.59 | 2.30 | 0 to 10 | 101 (9.7) |  |
| Participant PAI current | 997 | 25.06 | 3.70 | 5 to 30 | 41 (3.9) |  |
| Participant CCI number of non-dementia conditions | 1004 | 1.72 | 1.56 | 0 to 9 | 34 (3.3) |  |
| FAQ | 964 | 17.58 | 8.57 | 0 to 33 | 74 (7.1) |  |
| NPI-Q number of symptoms | 987 | 3.53 | 2.47 | 0 to 11 | 51 (4.9) |  |
| RSS | 975 | 19.34 | 9.73 | 0 to 51 | 63 (6.1) |  |
| Caregiver CESD-R | 973 | 7.07 | 7.60 | 0 to 48 | 65 (6.3) |  |
| Caregiver PAI current | 1015 | 23.34 | 4.73 | 7 to 30 | 23 (2.2) |  |
| Caregiver CCI number of conditions | 966 | 1.46 | 1.43 | 0 to 9 | 72 (6.9) |  |
| Age difference | 1038 | 2.65 | 5.37 | -13 to 29 | - |  |

AD Alzheimer’s disease, VaD Vascular dementia, FTD Frontotemporal dementia, PDD Parkinson’s disease dementia, DLB Dementia with Lewy bodies, MMSE Mini-Mental State Examination, GDS-10 Geriatric Depression Scale-10, PAI Positive Affect Index, CCI Charlson Comorbidity Index, FAQ Functional Activities Questionnaire, NPI-Q Neuropsychiatric Inventory Questionnaire, RSS Relative Stress Scale, CESD-R Center for Epidemiologic Studies Depression Scale-Revised

**Supplementary Table 2. Sex differences between variables**

|  | **People with dementia** | |
| --- | --- | --- |
| **Participant variables** | **Male** | **Female** |
| Participant age group (n, %) |  |  |
| <65 | 58 (8.5) | 36 (10.2) |
| 65-69 | 85 (12.4) | 61 (17.3) |
| 70-74 | 132 (19.3) | 73 (20.7) |
| 75-79 | 171 (25.0) | 94 (26.6) |
| 80+ | 239 (34.9) | 89 (25.2) |
| Dementia type (n, %) |  |  |
| AD | 363 (53.0) | 219 (62.0) |
| VaD | 79 (11.5) | 28 (7.9) |
| Mixed AD/VaD | 134 (19.6) | 65 (18.4) |
| FTD | 31 (4.5) | 12 (3.4) |
| PDD | 29 (4.2) | 10 (2.8) |
| DLB | 35 (5.1) | 4 (1.1) |
| Unspecified/Other | 14 (2.0) | 15 (4.2) |
| Time since diagnosis (n, %) |  |  |
| <1 year | 325 (51.9) | 184 (55.1) |
| 1-2 years | 207 (33.1) | 106 (31.7) |
| 3+ years | 94 (15.0) | 44 (13.2) |
| MMSE (mean, SD) | 23.43 (3.71) | 22.56 (3.56) |
| Self-rated variables (mean, SD) |  |  |
| GDS-10 | 2.61 (2.27) | 2.56 (2.37) |
| Participant PAI current | 25.05 (3.64) | 25.08 (3.82) |
| Participant CCI number of conditions | 1.84 (1.60) | 1.50 (1.44) |
| Informant-rated variables (mean, SD) |  |  |
| FAQ | 17.92 (8.73) | 16.93 (8.23) |
| NPI-Q number of symptoms | 3.74 (2.50) | 3.11 (2.37) |
|  | **Caregivers** | |
| **Caregiver variables** | **Male** | **Female** |
| Caregiver age group (n, %) |  |  |
| <65 | 31 (8.9) | 125 (18.1) |
| 65-69 | 54 (15.6) | 144 (20.8) |
| 70-74 | 80 (23.1) | 178 (25.8) |
| 75-79 | 64 (18.4) | 152 (22.0) |
| 80+ | 118 (34.0) | 92 (13.3) |
| Caregiver education (n, %) |  |  |
| No qualifications | 74 (21.5) | 181 (26.2) |
| School leaving certificate at age 16 | 56 (16.3) | 177 (25.7) |
| School leaving certificate at age 18 | 125 (36.3) | 184 (26.7) |
| University | 89 (25.9) | 148 (21.4) |
| Caregiver self-rated variables (mean, SD) |  |  |
| RSS | 16.32 (8.90) | 20.80 (9.79) |
| Caregiver CESD-R | 4.76 (5.98) | 8.21 (8.04) |
| Caregiver CCI number of conditions | 1.50 (1.33) | 1.45 (1.47) |
| Caregiver PAI current | 24.80 (4.26) | 22.81 (4.80) |
| Caregiver Self-rated health (n, %) |  |  |
| Poor/Very poor | 17 (4.9) | 55 (8.1) |
| Fair | 75 (21.8) | 171 (25.1) |
| Good | 127 (36.9) | 246 (36.2) |
| Very good | 91 (26.5) | 158 (23.2) |
| Excellent | 34 (9.9) | 50 (7.4) |
| Hours of caregiving per day (n, %) |  |  |
| <1 hour | 69 (20.3) | 137 (20.0) |
| 1-10 hours | 140 (41.2) | 228 (33.3) |
| 10+ hours | 131 (38.5) | 319 (46.6) |
|  |  |  |
| **Dyadic variables** | **Male** | **Female** |
| Area deprivation* (n, %) |  |  |
| Q1 Most deprived | 54 (7.9) | 22 (6.2) |
| Q2 | 93 (13.6) | 46 (13.0) |
| Q3 | 144 (21.0) | 75 (21.2) |
| Q4 | 166 (24.2) | 99 (28.0) |
| Q5 Least deprived | 228 (33.3) | 111 (31.4) |

AD Alzheimer’s disease, VaD Vascular dementia, FTD Frontotemporal dementia, PDD Parkinson’s disease dementia, DLB Dementia with Lewy bodies, MMSE Mini-Mental State Examination, GDS-10 Geriatric Depression Scale-10, PAI Positive Affect Index, CCI Charlson Comorbidity Index, FAQ Functional Activities Questionnaire, NPI-Q Neuropsychiatric Inventory Questionnaire, RSS Relative Stress Scale, CESD-R Center for Epidemiologic Studies Depression Scale-Revised.

*Area deprivation applies to both people with dementia and their caregiver as all dyads are coresident, however the n and percentages of males and females in the table relate only to the people with dementia who took part in the study.

**Supplementary Figure 1.** RADIX scores for dyads with RADIX-Difference of zero (n=126 dyads)
